# Amputation and reamputation for dry gangrene of both lower extremities in with chronic kidney disease patients with calciphylaxis accompanied by multidrug-resistant bacterial infections: A case report and literature analysis

**DOI:** 10.1097/MD.0000000000047239

**Published:** 2026-01-16

**Authors:** Xingcheng Zhao, Huanhuan He, Ming Lu

**Affiliations:** a Department of Orthopedics, Shanghai Public Health Clinical Center, Shanghai, China.

**Keywords:** amputation, calciphylaxis, chronic kidney disease, dry gangrene, reamputation

## Abstract

**Rationale:**

Nephritis can further develop into chronic kidney disease (CKD). CKD with calciphylaxis is mainly manifested as necrotic ulcers of terminal tissues or even gangrene in the skin and distal limbs. Patients with CKD have a high risk of developing multidrug-resistant bacterial infections.

**Patient concerns:**

Our patient developed dry gangrene of both feet for 1 month, accompanied by complications, such as anemia, hypoproteinemia, and pulmonary infection. The patient had a history of CKD for 30 years, calciphylaxis for 5 years, and multidrug-resistant bacterial infection for 1 month.

**Diagnoses:**

The patient was diagnosed with atherosclerotic gangrene of the lower extremities (dry gangrene of both feet), multidrug-resistant bacterial infection, pulmonary infection, hypoalbuminemia, anemia (moderate), urinary tract infection, and CKD with calciphylaxis.

**Interventions:**

In this case, the patient underwent a clinical evaluation and was successfully resuscitated. After achieving clinical cure criteria for multidrug-resistant bacterial infection, the patient underwent a mid-calf amputation of both legs under general anesthesia. Half a year post-operation, due to a recurrence of multidrug-resistant bacterial infection, the stumps of the amputated legs ulcerated with bone exposure. While treating multidrug-resistant infection, the patient was pharmacologically treated for CKD. In addition, debridement of the infected wounds and reamputation of both leg stumps were performed.

**Outcomes:**

Following the reamputation surgery, the patient underwent stump training for 3 months, and then walked on ground with prostheses.

**Lessons:**

Reflections prompted by this case: a textbook approach to treatment (standardized, systematic treatment strategy) is crucial. In this case, the primary objective was to save the patient’s life, concurrently alleviating symptoms such as anemia and hypoproteinemia, rigorously combating multidrug-resistant bacterial infection, and performing a mid-calf amputation of both legs. Six months after operation, the recurrence of multidrug-resistant bacterial infection resulted in bone exposure at the stumps of both legs, and the patient had to undergo reamputation of both leg stumps.

## 1. Introduction

Dry gangrene of the foot is a serious condition caused by local tissue ischemic necrosis, mostly resulting from inflammation of the small arteries in the foot due to complex factors. Clinically, it manifests as blackening of the skin starting from the ends of the toes, along with dry and hardened tissue changes. Dry gangrene commonly develops in patients with diabetes^[1]^ and thromboembolic arterial occlusive vasculitis.^[2]^ There are also reports on rare disease- and even drug-induced dry gangrene. For example, dry gangrene may occur in patients with sickle cell disease,^[3]^ type 1 cryoglobulinemia combined with lymphoplasmacytic lymphoma,^[4]^ and chronic myeloid leukemia undergoing mild amputation following chronic limb-threatening ischemia revascularization: finger ischemia and gangrene,^[5]^ and rare severe community-acquired bloodstream infections.^[6]^

Patients with long-term chronic kidney disease (CKD) often experience recurrent episodes and gradual worsening, and even develop calciphylaxis, characterized by small artery thrombosis, arteriosclerosis, and luminal stenosis/occlusion. These conditions lead to ischemic necrosis of the distal extremities, resulting in dry gangrene. Long-term follow-up visits have also revealed that patients with CKD are at a high risk of multidrug-resistant bacterial infections.^[7]^ These infections commonly include urinary tract infections, pneumonia, skin and soft tissue infections, or hematogenous infection, and even bacteremia or sepsis. Infections with multidrug-resistant bacteria in CKD patients are often life-threatening, and therefore, active life-saving is a key component.

Herein, our treatment team reported a case of CKD accompanied by dry gangrene in both feet and multiple drug-resistant bacterial infections. After life-saving measures, anti-infection treatment, and CKD management, the patient underwent mid-calf amputation of the both lower limbs and reamputation surgeries. Now, the patient can successfully walk with prostheses. The details are as follows:

## 2. Clinical data

**Figure 1.**
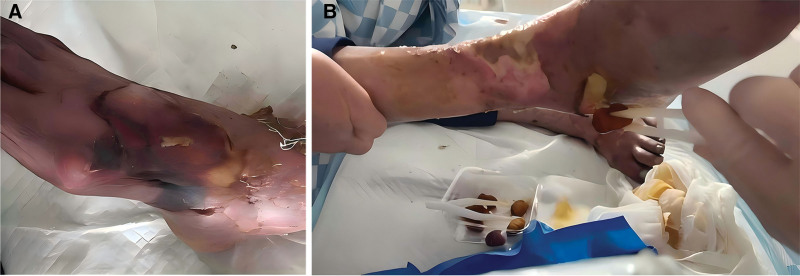
Early onset of the disease, large-area tension blisters began to appear 2 weeks after swelling in both feet, as well as severe pain in both feet (A: right foot, B: left foot). In the early stages of the disease, the skin exhibited reticular purpura and violaceous macules, followed by the development of ulcers.

**Figure 2.**
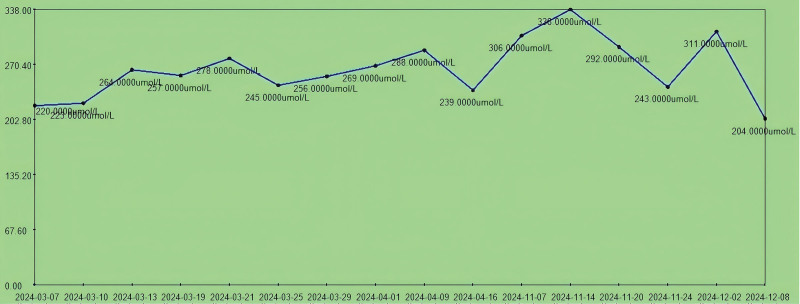
The trend chart of uric acid level during hospitalization. The patient’s uric acid was controlled at 220 to 338.0 µmol/L during hospitalization.

A 74-year-old male patient (Li XX) presented with bilateral foot redness, swelling, and pain accompanied by the formation of tense blisters (Fig. 1) 3 months ago without identifiable triggers. Initial diagnosis at the local hospital included: CKD, hyperuricemia, hyperhomocysteinemia, bilateral lower limb edema, and hypertension. Therapeutic interventions, including renal function improvement, urate-lowering therapy, anti-inflammatory agents, and microcirculatory enhancement, were given. Serological testing showed that the level of uric acid gradually decreased to a normal level (Fig. 2). However, 2 weeks later, clinical progression was noted: persistent pedal edema of both feet without significant relief, and the appearance of dry gangrene symptoms such as blackening of both toes appeared. There was pronounced swelling around both calves and ankles, with redness and swelling on the dorsum of both feet. Both toes exhibited a blackish-purple discoloration, and the bilateral dorsal arteries pulsed weakly. Additionally, skin lesions were present on the right foot dorsum.

Urine bacterial culture indicated the presence of *Enterococcus faecium* in the urine. Microbial culture of the infected foci on the foot dorsum indicated the presence of *Staphylococcus aureus*, a multidrug-resistant strain, which was sensitive to vancomycin, tigecycline, nitrofurantoin, rifampicin, linezolid, and gentamicin. The patient had a 30-year history of CKD, a 10-year history of hyperuricemia, and a 5-year history of recurrent bilateral foot swelling.

**Figure 3.**
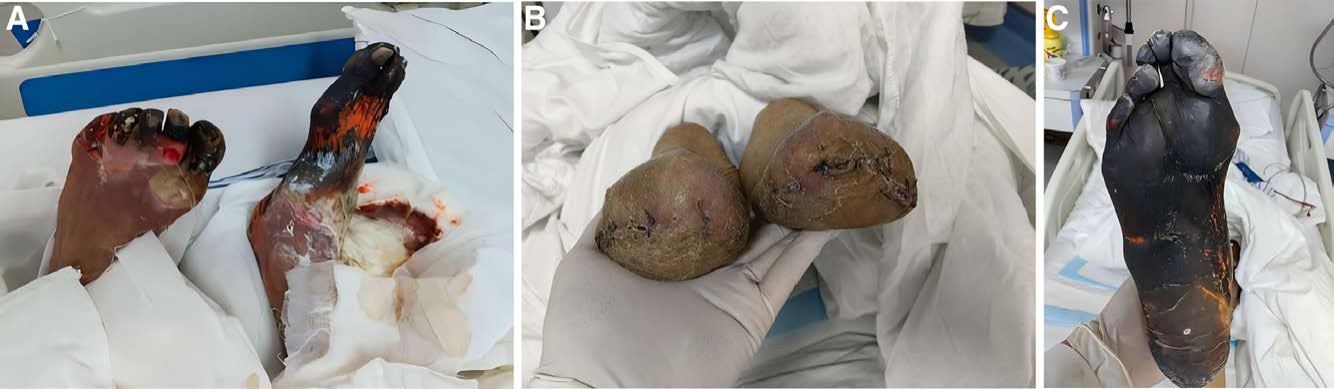
The patient with gangrene of both feet (A: front; C: right plantar), after bilateral calf amputation (B).

The patient was admitted with lethargy and hypothermia. The skin of the left foot toes 1 to 5 was dry and blackened, with ulcerative infection at the distal metatarsal. Red and white drug residues were visible on the skin, and proximal skin sensation remained intact. The left ankle could move voluntarily, and the dorsalis pedis artery pulsed weakly. The right foot exhibited dry, darkened skin distal to the ankle joint resembling dead tree branches, with proximal skin infection and exfoliation. The whole plantar surface of the right foot became blackened and dry (Fig. 3). The right ankle could be moved voluntarily and proximal skin sensation was fair. Bilateral calf skin temperature was significantly lower than that of the bilateral thighs. Both hands were swollen, but maintained flexion and extension mobility. The right shoulder was stiff and unable to be lifted. The patient was extremely emaciated, with edema of the prepuce and scrotum.

This study was conducted in compliance with the *Declaration of Helsinki* and the relevant ethical requirements for research set forth by the Shanghai Public Health Clinical Center. The hospital ethics approval number is [2020]2020-S12-02, with the approval date of July 24, 2020. Patients and their families have been fully informed about the entire study process, potential risks, and possible outcomes. Informed consent has been obtained from both patients and their families.

### 2.1. Therapeutic procedures

**Figure 4.**
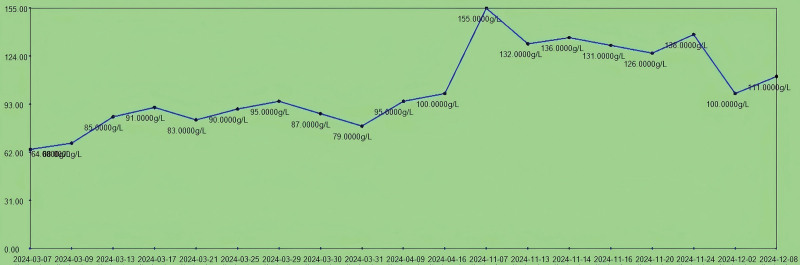
The trend chart of hemoglobin levels (130.00–175.00 g/L) in the patient during hospitalization.

**Figure 5.**
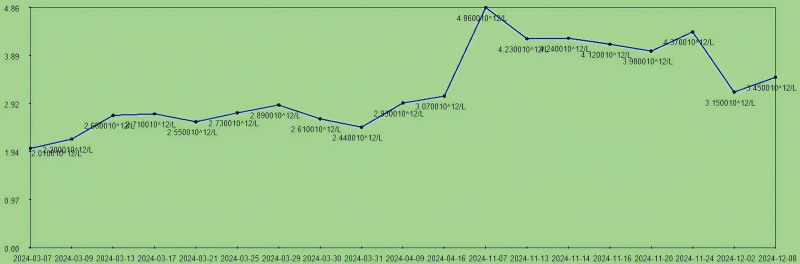
The trend chart of erythrocyte counts (4.30–5.80 × 10^12^/L) during hospitalization.

**Figure 6.**
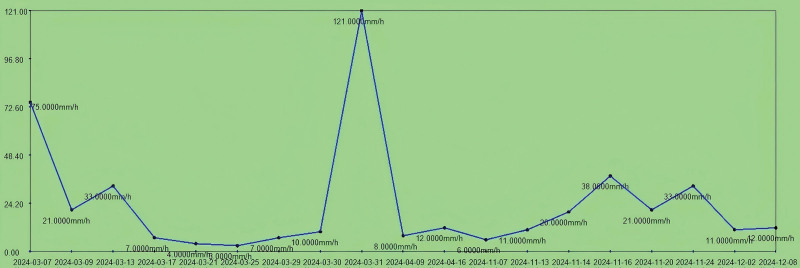
The trend chart of erythrocyte sedimentation rate (0–15 mm/h) during hospitalization.

**Figure 7.**
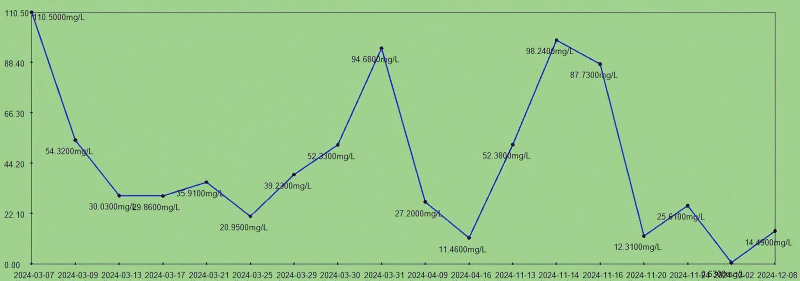
The trend chart of C-reactive protein (0.00–10.00 mg/L) during hospitalization.

**Figure 8.**
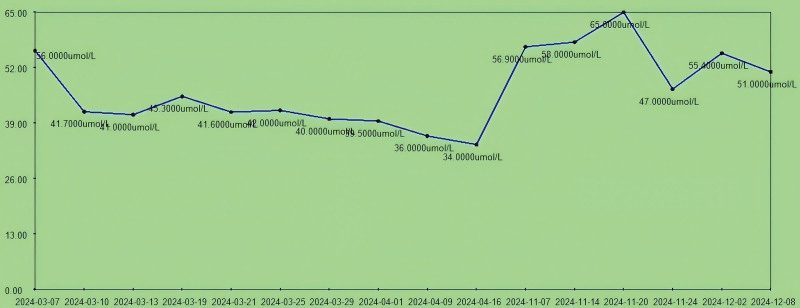
The trend chart of creatinine level (57.00–111.00 µmol/L) during hospitalization. The patient’s creatinine was consistently controlled at 56-34-65-51 µmol/L during hospitalization.

**Figure 9.**
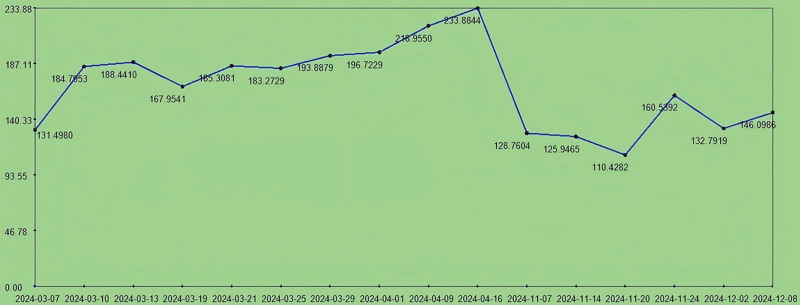
The trend chart of creatinine clearance rate (> 90 mL/[min·1.73 m^2^]) during hospitalization.

Upon admission, the patient underwent a thorough clinical examination. Definitive diagnoses were made: Atherosclerotic gangrene of both lower limbs (dry gangrene of both feet), soft tissue infection of both feet, pulmonary infection, urinary tract infection, atrial fibrillation, hypoproteinemia, swelling of both upper limbs, and moderate anemia. The patient was classified as critically ill, and life-saving measures were immediately initiated, including correction of acidosis, fluid and electrolyte balance, and anti-infective therapy. Key laboratory test results after admission are shown in Figures 2 and 4–9.

After the patient’s vital signs were stabilized and the multi-drug-resistant bacterial infection was clinically cured, bilateral mid-calf amputation was performed under general anesthesia. The surgical process was completed smoothly. The wound healed in stage 1 after the operation, reaching the clinical cure standard, and the patient was discharged with instructions for follow-up observation at a local hospital outpatient clinic.

**Figure 10.**
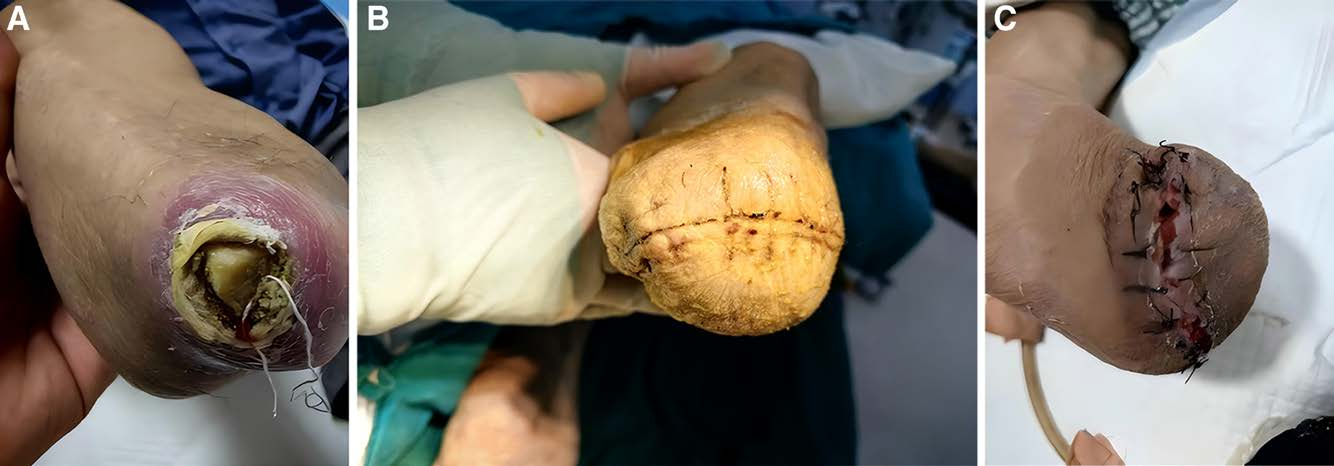
Stump infection with exposed bone (A) after bilateral calf amputation (B: right leg; C: left leg).

**Figure 11.**
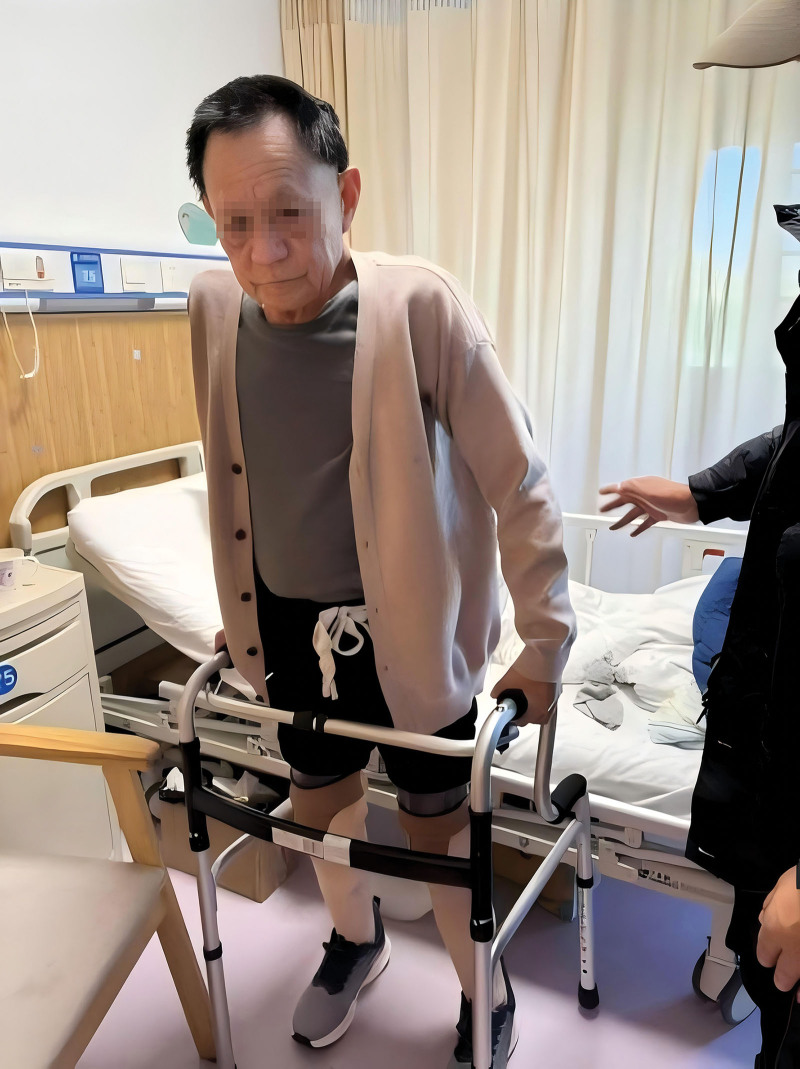
Diagram of the patient walking with prosthesis.

Six months later, the patient experienced a recurrence of multidrug-resistant bacterial infection, with ulceration of the bilateral lower limb amputation stumps and exposed bone (Fig. 10A). Treatment for multidrug-resistant bacterial infection was taken, along with active management and stabilization of CKD progression. Debridement of the infected stumps and reamputation of both lower limbs were performed (Fig. 10B, C). Three months after the reamputation surgery, the patient was able to walk with a prosthesis (Fig. 11). The clinical outcome was satisfactory.

Upon admission, the patient presented with anemia and critical values for infectious indicators, indicating multidrug-resistant bacterial infection (severe infection; Figs. 6 and 7). As shown in Figure 4, the patient presented with severe anemia and hypoproteinemia upon admission. After transfusion of 4 units of freshly suspended erythrocytes, symptoms of anemia were partially corrected (hemoglobin increased to 91 g/L), and the multidrug-resistant bacterial infection was under control. Following the amputation surgery (March 17, 2024), the patient’s anemia was further managed (hemoglobin was 100 g/L at the first discharge). The amputation risk in this case was significantly associated with anemia and hypoproteinemia, consistent with the findings reported.^[8]^

Upon admission, the patient presented with severe anemia and hypoproteinemia. After transfusion of 4 units (600 mL) of freshly suspended erythrocytes, the patient’s anemia symptoms were partially corrected (erythrocytes increased from 2.0 × 10^12^/L to 2.89 × 10^12^/L), while the multidrug-resistant bacterial infection was under control. The patient’s anemia symptoms were further controlled after the amputation surgery (March 17, 2024; erythrocytes were 3.07 × 10^12^/L at the first discharge). The number of erythrocytes was 4.86 × 10^12^/L at readmission and the patient’s red blood cells and hemoglobin levels were stabilized during the second admission (Fig. 5). This indicates that the treatment results for anemia were stable at the first admission.Upon admission, the patient’s erythrocyte sedimentation rate (ESR) was 75 mm/h. After treatment for multidrug-resistant bacterial infection, the ESR decreased to 4.4 mm/h and remained stable between 4 mm/h and 10 mm/h postamputation. On March 31, 2024, the patient experienced sudden high fever, coughing, and expectoration, suggestive of a pulmonary infection. Symptomatic treatment was given, and the body temperature returned to normal. Inflammatory markers such as ESR and C-reactive protein (CRP; Fig. 6) gradually decreased to normal levels. On November 16, 2024, the patient was readmitted due to a recurrence of multidrug-resistant bacterial infection, with an ESR of 38 mm/h. Infectious ulcers reappeared at the amputation stumps, accompanied by exposed bone. Following treatment for multidrug-resistant bacterial infection and reamputation of the stumps, the patient’s ESR returned to normal ranges (the ESR decreased to 11 mm/h on December 2, 2024). Upon admission, the patient’s CRP level was 110.5 mg/L. After treatment for multidrug-resistant bacterial infection, the CRP decreased to 20 mg/L and remained stable between 30 mg/L and 20 mg/L after amputation. On March 31, 2024, the patient experienced sudden high fever, coughing, and expectoration, suggestive of a pulmonary infection (the CRP was 94.68 mg/L). Symptomatic treatment was given, and the body temperature returned to normal, with the CRP gradually decreasing to normal levels (27 mg/L). On November 16, 2024, the patient was readmitted due to a recurrence of multidrug-resistant bacterial infection, with a CRP level of 98.24 mg/L. Infectious ulcers reappeared at the amputation stumps, accompanied by exposed bone. Following treatment for multidrug-resistant bacterial infection and reamputation of the stumps, the patient’s CRP returned to normal ranges (the CRP was 12 mg/L on December 2, 2024; Fig. 7). The changes in the patient’s ESR and CRP are consistent with those previously reported.^[9,10]^

Although the patient’s 24-hour creatinine clearance rate decreased during hospitalization, it remained above the normal range (Fig. 9). This result was considered to be due to the patient’s long-term CKD with reduced renal function or renal insufficiency. Therefore, improving renal function and controlling the progression of CKD after surgery remain long-term tasks.

## 3. Discussion and literature analysis

Calciphylaxis is a rare disease that primarily arises in individuals with CKD who are on dialysis. This disorder results from the progressive and chronic calcification of small arteries, which subsequently leads to thrombosis of the residual lumen.^[7]^ The clinical manifestations of the disease are pruritic cutaneous laminar erythema, ischemic purpura and skin necrosis with severe pain. The patient had a 20-year history of CKD and presented with renal insufficiency, hypoproteinemia, low phosphorus and calcium levels, hyperuricemia, hyperhomocysteinemia, and atherosclerosis upon admission.

Calciphylaxis typically presents in 2 forms that can occur simultaneously: In the first stage, the condition is often insidious and usually asymptomatic; and sometimes it may present with pruritus and patchy erythema of the skin, with a mortality rate of approximately 30%. In the second stage, the disease progresses rapidly and is characterized by ischemic purpura with severe pain, followed by skin ulceration and necrosis. In this stage, the mortality rate is about 80%.^[7]^

Histological examination of patients with calciphylaxis reveals concurrent medial calcification, thrombosis, and involvement of the intimal elastic layer. Skin lesions can be displaced at distal (associated with better prognosis) or proximal (associated with worse prognosis) ends, but they may also appear in other non-subcutaneous areas. Risk factors for calciphylaxis in patients with CKD include female, diabetes, obesity, malnutrition, disorders of bone mineral metabolism, and the use of vitamin K antagonists.^[7]^ Diagnosis is primarily based on clinical manifestations, and imaging examination may be used as an adjunct to skin biopsy in cases where the diagnosis is uncertain. Early treatment is crucial to prevent disease progression, control the spread of infection, and reduce morbidity and mortality. The optimal treatment plan involves a combination of ulcer wound care, infection prevention, elimination of high-risk factors, administration of appropriate medications, and close medical follow-up.

**Figure 12.**
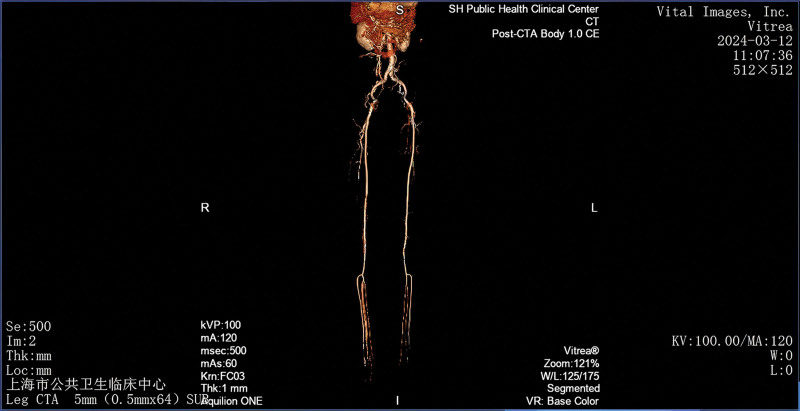
Computed tomography angiography (CTA) of the lower abdomen, pelvis, and lower extremities (iliac artery CTA + lower extremity CTA; March 12, 2024), indicating that multiple calcified plaques with luminal stenosis were visible in the bilateral common iliac arteries, a limited true-false lumen of approximately 0.9 cm was formed in the left common iliac artery, and noncalcified plaque luminal stenosis was scattered in the remaining external iliac arteries on both sides, distal femoral artery, popliteal artery, anterior and posterior tibial arteries, and peroneal artery.

CKD patients with calciphylaxis who have developed dry gangrene of both lower limbs often need to be differentiated from the following conditions: lower limb arteriosclerosis obliterans (in patients with lower limb arterial embolism, the affected limb may experience sudden pain, numbness, coldness, and loss of arterial pulsation below the occlusion site; these patients often have a history of conditions such as atrial fibrillation or rheumatic heart disease, with a relatively acute onset; color Doppler ultrasound and arteriography can aid in differentiation); thromboangiitis obliterans (it is more common in young and middle-aged males, and over 90% of patients have a history of smoking; it mainly affects arteries of the lower limbs, such as the dorsal pedal artery, posterior tibial artery, popliteal artery, or femoral artery; about 40% of patients experience recurrent migratory thrombophlebitis in the lower legs and feet during the early stages or course of the disease); and diabetic foot (diabetic foot is more common in diabetic patients, presenting with foot pain, ulceration, color changes, and sometimes accompanied by hypersensitivity of peripheral nerves; angiography may reveal changes in the peripheral arteries of the lower limbs, which can aid in differentiation). In the present case, the patient also demonstrated significant alterations in the peripheral arteries of the lower limbs. A subsequent computed tomography angiography of the lower abdomen, pelvis, and lower extremities, performed on March 12, 2024 (Fig. 12), revealed multiple calcified plaques with luminal stenosis in the bilateral common iliac arteries. A limited true-false lumen, measuring approximately 0.9 cm, was observed in the left common iliac artery. Furthermore, noncalcified plaques causing luminal stenosis were observed in the bilateral external iliac arteries, distal femoral arteries, popliteal arteries, anterior and posterior tibial arteries, and peroneal arteries. These imaging findings are consistent with the widespread vascular calcification that is commonly observed in patients with CKD and calciphylaxis.

Additionally, in the treatment of CKD patients with calciphylaxis leading to dry gangrene of both lower limbs, the primary focus is on controlling calcium and phosphorus metabolism and optimizing parathyroid hormone levels. Pharmacological treatments mainly include: sodium thiosulfate, which chelates calcium ions and exerts an antioxidant mechanism, reducing calcium deposition; bisphosphonates, which can inhibit the formation of calcium phosphate in the treatment of calciphylaxis; analgesic and anti-infection treatments; and surgical treatments, primarily involving amputation.

Regarding “Multidrug-Resistant Organism Management”: Multidrug resistance primarily refers to bacteria exhibiting simultaneous resistance to 3 or more clinically used classes of antimicrobial agents. Examples include: methicillin-resistant *S aureus*; vancomycin-resistant *Enterococcus*; extended-spectrum β-lactamases; carbapenem-resistant *Acinetobacter baumannii*, *Pseudomonas aeruginosa*, and Enterobacteriaceae resistant to cephalosporins, aminoglycosides, and fluoroquinolones. Such bacteria are classified as multidrug-resistant bacteria. Biological management primarily includes: enhancing healthcare workers’ hand hygiene compliance; strictly implementing disinfection and isolation measures; rigorously executing aseptic techniques; rational use of antimicrobial agents; strengthening cleaning and disinfection procedures; and establishing and improving surveillance systems for multidrug-resistant bacteria.

**Figure 13.**
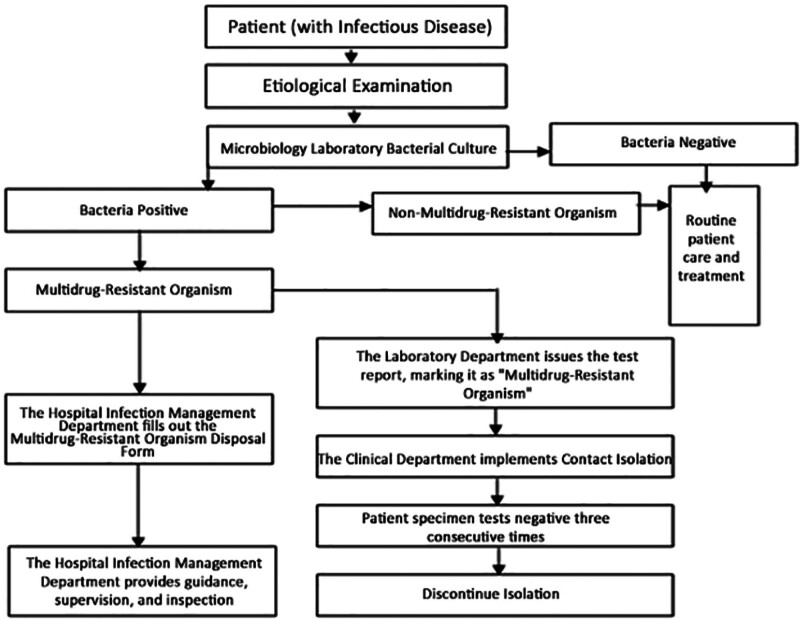
The management process for multidrug resistance.

The management process for multidrug resistance is as follows (Fig. 13):

Patients with CKD are at high risk of multidrug-resistant bacterial infection.^[11]^ Treating and controlling multidrug-resistant bacterial infections in such patients is an important part of improving patients’ outcomes and survival (Figs. 6 and 7).^[1]^

The progression of dry gangrene in calciphylaxis patient with CKD is exacerbated by multidrug-resistant bacterial infection.^[11]^ In this case, the patient initially presented with bilateral foot swelling and low skin temperature at the local hospital, and cyanosis of the plantar surfaces progressed to dry gangrene of the toes within 5 to 7 days. Over the subsequent month, the condition deteriorated with complete gangrenous involvement of all 5 right toes, formation of tension blisters on the medial right foot surface, erythematous swelling of the right foot and weakened dorsalis pedis arterial pulsation. The left foot presented with gangrene of the distal thumb and exhibited less severe edema compared with the right, with preserved dorsalis pedis arterial pulsation. Within approximately 3 months, both feet were completely blackened and dry, subsequently developing dry gangrene.

In the United States, multidrug-resistant bacterial infections mostly result from urinary tract infections, pneumonia, and sepsis.^[12]^ A research study from Denmark^[13]^ showed that the risk factors for reamputation after major lower limb amputation were smoking, advanced age, hyoxemia, diabetes mellitus, renal insufficiency, and a history of revascularization. Infection is the second most common cause of death in patients with end-stage renal disease.^[11]^

Major lower limb amputation is defined as the removal of the part of the lower limb above the ankle joint and is categorized as hip disarticulation, transfemoral amputation, knee disarticulation, and transtibial amputation. Limb-preserving amputations aim to optimize functional outcomes for the amputee, although the risk of reamputation may be higher for below-knee amputations.^[14–16]^

It has been reported that lower hemoglobin levels and higher levels of CRP, neutrophil to lymphocyte ratio and platelet to lymphocyte ratio in patients with diabetic foot may be associated with higher risk of lower limb amputation.^[8]^ Observational studies in Nigeria have shown that anemia is associated with delayed wound healing, amputation and higher mortality.^[17,18]^

## 4. Prognosis management

In this case, changes in the levels of hemoglobin, CRP, neutrophil to lymphocyte ratio and platelet to lymphocyte ratio were positively correlated with reamputation. Therefore, we closely observed the changes in the above key indexes, and made timely adjustments to correct the degree of anemia during the long-term follow-up. We also controlled infectious conditions in the patient, to minimize the recurrence of CKD and multidrug-resistant bacterial infections, ensuring the patient’s quality of life after reamputation.

Clinical reports have documented severe cases of hydroxyurea-induced gangrene in patients with chronic myeloid leukemia, but not in patients with sickle cell disease who typically receive long-term hydroxyurea treatment.^[10,17]^ Hydroxyurea is a standard therapeutic agent for preventing vasoocclusive crises in sickle cell disease and can lead to skin reactions, including pigmentation changes, dryness, and hair loss, which usually resolve after drug withdrawal.^[15,16,18]^ However, the pathogenesis of hydroxyurea-induced skin complications in sickle cell disease remains unclear.^[17]^

It is certain that the treatment of vasoocclusive disease is highly challenging, which is attributed to a multitude of factors. Therefore, it is difficult to establish a unified treatment approach. In this case, due to the unstable control of CKD, the primary focus was on aggressively treating the underlying disease, striving to eliminate factors contributing to vascular occlusion, and actively preventing the risk of reamputation.

In addition to the aforementioned treatments, it is necessary to consider multidrug-resistant bacterial infection as a factor contributing to the risk of reamputation in this case. Furthermore, the potential risks associated with prophylactic pharmacological interventions should be considered, as reported previously: severe cases of hydroxyurea-induced gangrene have been reported in patients with chronic myeloid leukemia.^[3]^ Treatment and real-time monitoring are equally important. In this case, we emphasized the surveillance of key indicators and vital signs during treatment to ensure the patient’s therapeutic efficacy.

Clinical timeline and sequence of therapeutic interventions:

Phase I:

1. Day 0: The patient was admitted and received a definitive diagnosis: Atherosclerotic gangrene of both lower limbs (dry gangrene of both feet), soft tissue infection of both feet, pulmonary infection, urinary tract infection, atrial fibrillation, hypoalbuminemia, swelling of both upper limbs, and anemia (moderate).

Therapeutic interventions: Controlling CKD and improving renal function; anti-multidrug-resistant bacterial infection therapy; internal environment stabilization therapy: blood transfusion, human albumin infusion, potassium supplementation, calcium supplementation, acidosis correction, and maintenance of electrolyte balance.

Outcomes: The infection was effectively controlled, with 3 consecutive negative bacterial culture results.

2. Day 30: Surgical intervention: bilateral mid-calf amputation (while continuing anti-infective therapy).

Outcomes: Following amputation surgery, the surgical wound healed in the primary stage. Concurrently, 3 consecutive follow-up examinations showed negative results for infection markers.

Phase II:

3. Day 290: Six months postoperatively, the patient experienced a recurrence of multidrug-resistant bacterial infection, resulting in exposed bone at the residual stumps following bilateral below-knee amputations.

Therapeutic interventions: Anti-infective therapy was reinitiated to control CKD, improve renal function, and stabilize the internal environment.

Outcomes: The infection was effectively controlled, with 3 consecutive negative bacterial culture results.

4. Day 299: Reamputation surgery.

Outcomes: Following amputation surgery, the surgical wounds healed by primary intention. Concurrently, 3 consecutive tests for infection markers were negative.

**Figure 14.**
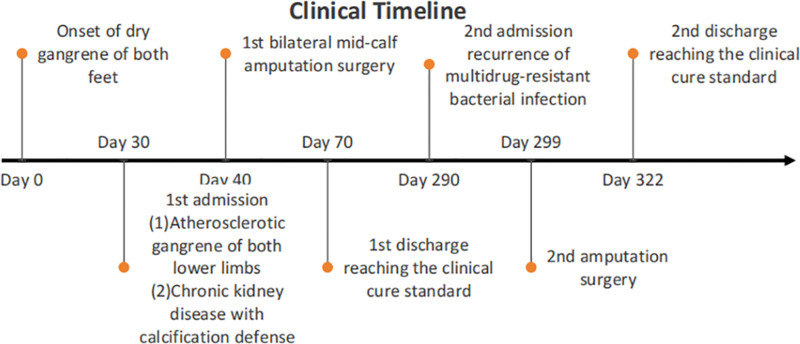
Clinical timeline.

5. Day 322: After 6 months of postoperative rehabilitation, the patient was able to walk with the prosthetic limb (see clinical timeline for details in Fig. 14).

For this patient, our treatment principles are as follows: to save the patient’s life by promptly restoring and maintaining vital signs, actively treating and controlling the underlying disease (CKD), effectively controlling multidrug-resistant bacterial infections, and timely performing bilateral calf amputations to further eliminate the source of infection and control its spread. However, both multidrug-resistant bacterial infections and CKD are conditions that require long-term treatment and follow-up. Therefore, this patient still needs regular monitoring of various indicators (inflammatory markers, renal function indicators, and vital signs) and timely symptomatic treatment and correction to ensure normal vital signs and maintain the current quality of life.

Short patient statement: I have been suffering from CKD for over 30 years. Throughout the progression of the disease, I have endured much pain caused by the disease itself, and even developed complications such as hypoproteinemia and anemia. The distress of poor treatment outcomes and the torment of the disease, especially the recent worsening of calciphylaxis from CKD, directly led to dry gangrene in both of my lower limbs. The intense pain and the agony of being unable to care for myself made me lose the courage and confidence to live, and I no longer had the desire to survive. However, Dr Lu and her medical team provided me with meticulous, active, and effective treatment. After enduring a long 30-year illness, I saw significant treatment results in just 1 month, which rekindled my will to live, and I began to actively cooperate with the treatment. However, due to my personal habits and living environment being unsuitable for home-based treatment and recovery, my multidrug-resistant bacterial infection recurred, and the stumps after bilateral amputation became infected again, resulting in exposed bone at the stumps. After another surgical treatment by Dr Lu medical team and early postoperative rehabilitation, I was able to stand again with the help of prosthetics and return to my original life. But I understand that from now on, I must develop good hygiene habits, regularly review various infection indicators, and undergo anti-infection treatment when necessary to prevent the recurrence of multidrug-resistant bacterial infections.

## Author contributions

**Conceptualization:** Ming Lu.

**Data curation:** Xingcheng Zhao, Huanhuan He.

**Supervision:** Ming Lu.

**Writing – original draft:** Xingcheng Zhao.

**Writing – review & editing:** Ming Lu.
